# Early Treatment-Related Mortality in Acute Leukemia: A Real-World, Single-Center Study From a Resource-Limited Setting

**DOI:** 10.7759/cureus.111245

**Published:** 2026-06-21

**Authors:** Noran AlJafar, Aamer Mohammed, Talib Ali, Elaf Al-sulaitti

**Affiliations:** 1 Hematology, Gloucestershire Hospitals NHS Foundation Trust, Gloucestershire, GBR; 2 Clinical Hematology, College of Medicine, University of Thi-Qar, Nassiriyah, IRQ; 3 Microbiology, College of Medicine, University of Thi-Qar, Nassiriyah, IRQ; 4 Hematology, Sheffield Teaching Hospitals NHS Foundation Trust, Sheffield, GBR

**Keywords:** acute leukemia, acute promyelocytic leukemia, early death, induction chemotherapy, treatment-related mortality

## Abstract

Background and objectives

Treatment-related mortality (TRM) remains a significant barrier to successful outcomes in acute leukemia despite therapeutic advances. This study aimed to evaluate the frequency, clinical features, and risk factors associated with early TRM in a real-world cohort from a resource-limited setting, defined as death occurring within 30 days of treatment initiation. The primary aims of this study were to evaluate the incidence and timing of early death (ED) and TRM across different blood cancer classifications. The secondary aim was to identify the specific clinical causes, such as infectious complications, hemorrhagic events, or refractory disease, that drive these fatalities.

Methods

We retrospectively analyzed 155 patients aged 14 to 100 years diagnosed with acute myeloid leukemia (AML), acute lymphoblastic leukemia (ALL), acute promyelocytic leukemia (APL), or acute undifferentiated leukemia (AUL) between 2016 and 2025 at a single tertiary center in Iraq. Patients received standard treatment protocols, including the 3+7 regimen, UKALL XC, ATRA/ATO (all-trans retinoic acid and arsenic trioxide), hyper-CVAD (hyper-fractionated cyclophosphamide, vincristine, adriamycin, and dexamethasone), azacitidine, ATRA/idarubicin, and others.

Results

The overall TRM rate was 9.0% (14/155). APL patients demonstrated the highest mortality at 28.6% (8/28), followed by ALL at 5.1% (3/59), and AML at 4.5% (3/66). Female patients accounted for 78.6% of deaths. The principal causes were sepsis (42.9%), differentiation syndrome (28.6%), intracranial hemorrhage (21.4%), and alveolar hemorrhage (7.1%). No statistically significant differences in clinical parameters were observed between patients with ED and those without ED.

Conclusions

TRM rates remain substantial, particularly among APL patients, with infection representing the predominant cause. These real-world findings highlight the need for early risk recognition, rapid supportive care, and improved treatment pathways for acute leukemia in resource-limited settings.

## Introduction

Acute leukemia comprises a heterogeneous group of hematological malignancies characterized by the uncontrolled proliferation of abnormal leukocytes in the bone marrow and peripheral blood. Acute promyelocytic leukemia (APL) is a distinct subtype of acute myeloid leukemia (AML) characterized by the accumulation of abnormal promyelocytes harboring the pathognomonic t(15;17) translocation, which results in the PML-RARA fusion gene and subsequent blockade of myeloid differentiation [1]. APL accounts for approximately 10-15% of AML cases and typically presents in middle-aged adults [1,2].

Historically, APL was associated with extremely poor outcomes, with the majority of patients succumbing to hemorrhagic complications within weeks of diagnosis. The coagulopathy associated with APL, comprising disseminated intravascular coagulation, hyperfibrinolysis, and thrombocytopenia, renders patients particularly susceptible to life-threatening bleeding, with the central nervous system representing the most common site of fatal hemorrhage [3]. The introduction of all-trans retinoic acid (ATRA), which induces differentiation of leukemic promyelocytes, revolutionized APL treatment [4,5,6]. Subsequently, the addition of arsenic trioxide (ATO) has transformed APL into the most curable form of acute leukemia in adults, with long-term survival rates exceeding 90% in patients who survive the initial treatment period.

Despite these therapeutic advances, early death (ED) remains a significant cause of treatment failure in APL. Deaths occurring within the first 30 days of diagnosis are primarily attributable to hemorrhagic complications, differentiation syndrome, and sepsis [7,8,9,10]. Differentiation syndrome, occurring in 5-25% of patients receiving ATRA or ATO, is a potentially fatal complication characterized by fever, respiratory distress, pulmonary infiltrates, pleural and pericardial effusions, hypotension, and acute renal failure [11]. Patients with hyperleukocytosis (white blood cell count >10 × 10⁹/L) at diagnosis are at the highest risk of this complication.

In contrast to APL, AML, and acute lymphoblastic leukemia (ALL) demonstrate different epidemiological patterns and treatment-related complications. ALL predominantly affects children and young adults, whereas AML incidence increases with age. Despite substantial improvements in supportive care, patients with AML remain vulnerable to infectious complications during periods of treatment-induced cytopenia [11,12,13]. Previous studies have identified baseline neutropenia, lymphopenia, and monocytopenia as risk factors for febrile neutropenia and documented infections [14]. Understanding the patterns and predictors of TRM is essential for optimizing patient outcomes, informing treatment decisions, and allocating healthcare resources appropriately. The objective of this study was to evaluate the incidence, causes, and clinical parameters associated with early TRM in patients with different subtypes of acute leukemia treated in a real-world, single-center cohort from Iraq.

## Materials and methods

Study design and population

This single-center, retrospective, observational study was conducted using data from the Cancer Centre registry at Al-Hussein Teaching Hospital, Nassiriyah, Iraq. We included 155 consecutive patients aged 14-100 years diagnosed with AML, ALL, APL, or acute undifferentiated leukemia (AUL) between January 2016 and December 2025.

Treatment protocols

Patients were treated according to the following protocols: 3+7 regimen comprising daunorubicin/cytarabine, idarubicin/cytarabine or doxorubicin/cytarabine (n = 47); UKALL XC protocol (n = 44); ATRA/ATO (n = 21); hyper-CVAD (hyper-fractionated cyclophosphamide, vincristine, adriamycin, and dexamethasone) (n = 15); azacitidine (n=11); ATRA/idarubicin (n = 7); cytarabine monotherapy (n = 4); low-dose cytarabine (n = 3); vincristine pre-phase treatment (n = 2); and FLAG-IDA (fludarabine, cytarabine, G-CSF and idarubicin) (n = 1).

Data collection

Clinical and laboratory parameters collected included white blood cell (WBC) count, peripheral blood blast percentage, platelet count, hemoglobin concentration, and absolute neutrophil count (ANC). Medical records were reviewed to obtain demographic information, disease characteristics, treatment regimens, and outcomes, including adverse events and causes of death.

Definitions

TRM was defined as death occurring within 30 days of treatment initiation. Early death was used synonymously with TRM throughout this study.

Statistical analysis

Descriptive statistics are presented as counts, frequencies, proportions, means, standard deviations (SD), and ranges. Continuous variables are presented as mean ± SD with range, while categorical variables are presented as frequencies and percentages. Mortality rates were calculated and presented graphically. Figures were generated and exported in high-resolution format suitable for publication. Comparisons between patients with early death and those without early death were performed using the unpaired t-test for continuous variables. A p-value < 0.05 was considered statistically significant.

## Results

Patient characteristics

Of 155 patients with acute leukemia registered between January 2016 and December 2025, 59 (38.1%) had ALL, 66 (42.6%) had AML, 28 (18.1%) had APL, and two (1.3%) had AUL. The mean age at diagnosis was 36.8 years (range: 14-100 years). Mean ages for ALL, AML, and APL were 31.89 ± 16.97, 43.53 ± 18.11, and 32.06 ± 15.66 years, respectively, with ALL patients comprising the youngest cohort (Table 1).

**Table 1:** Patient characteristics by leukemia subtype ALL: acute lymphoblastic leukemia; AML: acute myeloid leukemia; APL: acute promyelocytic leukemia; AUL: acute undifferentiated leukemia; ANC: absolute neutrophil count; ATRA: all-trans retinoic acid; ATO: arsenic trioxide; SD: standard deviation; WBC: white blood cell

Parameter	ALL (n = 59)	AML (n = 66)	APL (n = 28)	AUL (n = 2)
Frequency, n (%)	59/155 (38.1%)	66/155 (42.6%)	28/155 (18.1%)	2/155 (1.3%)
Age, years				
Mean ± SD	31.89 ± 16.97	43.53 ± 18.11	32.06 ± 15.66	30 ± 0
Range	15–76	15–100	14–70	30
Sex, n (%)				
Male	36 (61.0%)	38 (57.6%)	9 (32.1%)	1 (50%)
Female	23 (39.0%)	28 (42.4%)	19 (67.9%)	1 (50%)
Male-to-female ratio	1.57:1	1.35:1	0.47:1	1:1
Hemoglobin, g/dL				
Mean ± SD	8.89 ± 3.77	7.94 ± 1.75	7.53 ± 1.82	7.9 ± 0
Range	4–33	3.6–16	4.8–12.4	7.9
WBC, ×10⁹/L				
Mean ± SD	55.11 ± 70.42	52.17 ± 54.61	29.41 ± 41.13	21.4 ± 22
Range	1.1–390	0.9–228	1.1–150	5.8–37
Peripheral blasts, %				
Mean ± SD	45.8 ± 34	41.1 ± 34	36.5 ± 35	33.5 ± 37
Range	0–91	0–98	0–97	7–60
ANC, ×10⁹/L				
Mean ± SD	4.78 ± 7.14	3.25 ± 4.16	1.95 ± 2.97	2.7 ± 1.41
Range	0–40	0.1–20	0–12	1.7–3.7
Platelets, ×10⁹/L				
Mean ± SD	56.28 ± 62.51	41.22 ± 43.73	38.41 ± 45.9	49.5 ± 21.9
Range	0.8–234	4–298	1–225	34–65
Treatment regimen, n (%)				
3+7	1 (1.7%)	46 (69.7%)	0	0
UKALL XC	44 (74.6%)	0	0	0
Hyper-CVAD	13 (22.0%)	0	1 (3.6%)	1 (50%)
ATRA/ATO	0	0	21 (75.0%)	0
ATRA/idarubicin	0	0	7 (25.0%)	0
Azacitidine	0	11 (16.7%)	0	0
Low-dose cytarabine	0	3 (4.5%)	0	0
Cytarabine	0	4 (6.1%)	0	0
FLAG-IDA	0	1 (1.5%)	0	0
VCR pre-phase	1 (1.7%)	0	0	1 (50%)
30-day mortality, n (%)	3 (5.1%)	3 (4.5%)	8 (28.6%)	0 (0%)
Cause of death, n (%)				
Sepsis	3 (100%)	3 (100%)	0	—
Differentiation syndrome	0	0	4 (50%)	—
Intracranial hemorrhage	0	0	3 (37.5%)	—
Alveolar hemorrhage	0	0	1 (12.5%)	

Of the total cohort, 84 (54.2%) were male, and 71 (45.8%) were female. Male-to-female ratios were as follows: 1.57:1 for ALL, 1.35:1 for AML, and 0.47:1 for APL, demonstrating male predominance in ALL and AML but female predominance in APL. The median WBC count was 48.66 × 10⁹/L overall, with values of 55.11 × 10⁹/L, 52.17 × 10⁹/L, and 29.41 × 10⁹/L for ALL, AML, and APL, respectively. Patients demonstrated low hemoglobin concentration (mean: 8.22 ± 2.74 g/dL), elevated peripheral blast percentage (mean: 41.9 ± 34%), and low platelet count (mean: 46.5 ± 52.09 × 10⁹/L), consistent with expected findings in acute leukemia.

Treatment-related mortality

**Figure 1:**
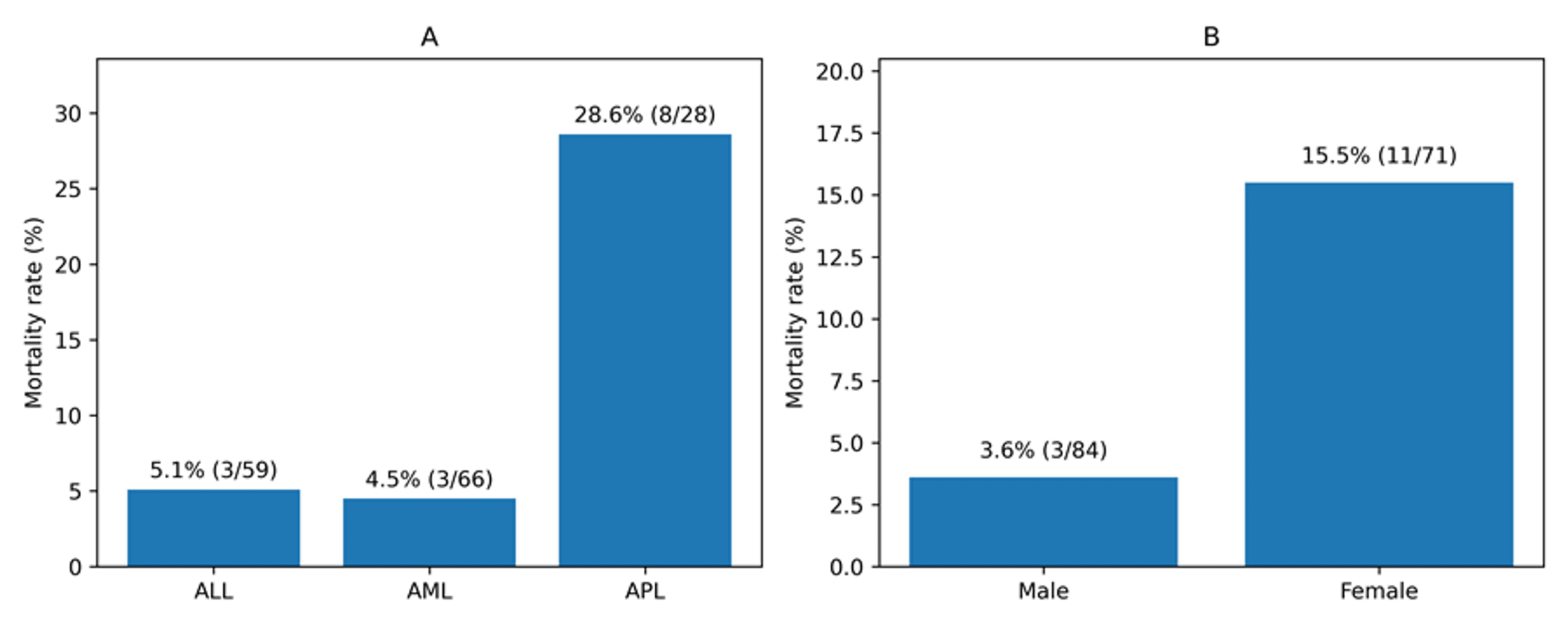
Mortality rates in acute leukemia by disease type and sex Panel A shows mortality rates by leukemia subtype represented as numbers (N) and percentages (%): 5.1% (3/59) in acute lymphoblastic leukemia, 4.5% (3/66) in acute myeloid leukemia, and 28.6% (8/28) in acute promyelocytic leukemia. Panel B shows mortality rates by sex, represented as numbers (N) and percentages (%): 3.6% (3/84) in male patients and 15.5% (11/71) in female patients ALL: acute lymphoblastic leukemia; AML: acute myeloid leukemia; APL: acute promyelocytic leukemia

The overall TRM rate was 9.0% (14/155). APL patients demonstrated the highest mortality rate at 28.6% (8/28), followed by ALL at 5.1% (3/59) and AML at 4.5% (3/66) (Figure 1A). The majority of deaths (8/14, 57.1%) occurred in APL patients, despite APL comprising only 18.1% of the study population.

Female patients accounted for 78.6% (11/14) of all deaths, with a mortality rate of 15.5% (11/71) compared with 3.6% (3/84) in males (Figure 1B).

The principal causes of TRM were sepsis (6/14, 42.9%), differentiation syndrome (4/14, 28.6%), intracranial hemorrhage (3/14, 21.4%), and alveolar hemorrhage (1/14, 7.1%) (Table 2).

**Table 2:** Characteristics of deceased patients Clinical and laboratory characteristics of 14 patients who died within 30 days of treatment initiation ALL: acute lymphoblastic leukemia; AML: acute myeloid leukemia; APL: acute promyelocytic leukemia; ANC: absolute neutrophil count; ATRA: all-trans retinoic acid; ATO: arsenic trioxide; SD: standard deviation; WBC: white blood cell

Characteristic	Value
Total deaths, n (%)	14/155 (9.0%)
Sex, n (%)	
Male	3 (21.4%)
Female	11 (78.6%)
Diagnosis, n (%)	
APL	8 (57.1%)
AML	3 (21.4%)
ALL	3 (21.4%)
Treatment regimen, n (%)	
ATRA/idarubicin	5 (35.7%)
3+7	4 (28.6%)
ATRA/ATO	3 (21.4%)
Hyper-CVAD	1 (7.1%)
UKALL XC	1 (7.1%)
Cause of death, n (%)	
Sepsis	6 (42.9%)
Differentiation syndrome	4 (28.6%)
Intracranial hemorrhage	3 (21.4%)
Alveolar hemorrhage	1 (7.1%)
Laboratory parameters, mean ± SD (range)	
Hemoglobin, g/dL	7.43 ± 1.33 (5.2–9.8)
WBC, ×10⁹/L	42.46 ± 21.31 (11–75)
Peripheral blasts, %	46.5 ± 31.57 (0–92)
ANC, ×10⁹/L	3.51 ± 4.23 (0.3–12)
Platelets, ×10⁹/L	26.61 ± 19.91 (6–65)
Age at diagnosis, years	38.07 ± 15.07 (18–70)

Sepsis was the sole cause of death in both ALL and AML patients, whereas APL deaths were attributable to differentiation syndrome (50%), intracranial hemorrhage (37.5%), and alveolar hemorrhage (12.5%). Deceased patients were treated according to the following regimens: ATRA/idarubicin (5/14, 35.7%), 3+7 (4/14, 28.6%), ATRA/ATO (3/14, 21.4%), hyper-CVAD (1/14, 7.1%), and UKALL XC (1/14, 7.1%). All three AML deaths occurred in patients receiving the 3+7 protocol.

Comparison of clinical parameters

Table 3 summarizes the comparison of hematological and demographic parameters between patients with and without early death. Deceased patients had lower mean hemoglobin (7.43 ± 1.33 vs. 8.30 ± 2.80 g/dL), lower mean platelet count (26.61 ± 19.91 vs. 48.61 ± 53.81 × 10⁹/L), and higher mean peripheral blast percentage (46.5 ± 31.57% vs. 41.5 ± 34.67%) compared with survivors. However, no statistically significant differences were observed in any of the clinical or demographic parameters analyzed (all p > 0.05).

**Table 3:** Comparison of early death vs. survivors Comparison of clinical and demographic parameters between patients with early death (≤30 days) and survivors. P-values were calculated using the unpaired t-test for continuous variables. A p-value < 0.05 was considered statistically significant ANC: absolute neutrophil count; SD: standard deviation; WBC: white blood cell

Parameter	Early death (n = 14)	No early death (n = 141)	P-value
Age, years			
Mean ± SD	38.07 ± 15.07	36.81 ± 18.27	0.806
Range	18–70	14–100	
Sex, n (%)			
Male	3 (21.4%)	81 (57.4%)	—
Female	11 (78.6%)	60 (42.6%)	—
Hemoglobin, g/dL			
Mean ± SD	7.43 ± 1.33	8.30 ± 2.80	0.240
Range	5.2–9.8	3.6–33	
WBC, ×10⁹/L			
Mean ± SD	42.46 ± 21.31	48.70 ± 61.52	0.905
Range	11–75	0.9–390	
Peripheral blasts, %			
Mean ± SD	46.5 ± 31.57	41.5 ± 34.67	0.597
Range	0–92	0–98	
ANC, ×10⁹/L			
Mean ± SD	3.51 ± 4.23	3.60 ± 5.49	0.945
Range	0.3–12	0–40	
Platelets, ×10⁹/L			
Mean ± SD	26.61 ± 19.91	48.61 ± 53.81	0.083
Range	6–65	0.8–298	

## Discussion

Early death remains a significant barrier to successful treatment outcomes in acute leukemia, particularly in real-world and resource-limited clinical settings. Despite the remarkable therapeutic advances that have transformed APL into a highly curable malignancy, ED rates ranging from 7% to 14% continue to be reported in developing countries [15,16]. Our study documented an overall TRM rate of 9.0%, consistent with these published figures.

In developed countries, complete remission rates in APL approach 95%, with disease-free survival at two years ranging between 87% and 97% [17]. However, studies from developing countries have reported substantially inferior outcomes, with complete remission rates of approximately 68%, induction mortality of 32%, and overall survival below 60% [18]. Our findings align with these observations, with APL demonstrating the highest mortality rate (28.6%) among all leukemia subtypes studied, accounting for 57.1% of all deaths despite comprising only 18.1% of the study population. This is consistent with previous reports from the Swedish Adult Acute Leukemia Registry and other population-based studies [10].

Sepsis emerged as the predominant cause of TRM in our cohort, accounting for 42.9% of all deaths and representing the sole cause of mortality in ALL and AML patients. This finding corroborates reports from other study groups identifying infection as a major contributor to early mortality, particularly in younger patients [19,20,21]. The high incidence of infectious complications underscores the critical importance of robust supportive care measures, including prompt initiation of broad-spectrum antimicrobial therapy, during the period of treatment-induced immunosuppression. Differentiation syndrome represented the second most common cause of TRM, accounting for 28.6% of deaths overall and 50% of APL deaths specifically. This potentially fatal complication of ATRA and ATO therapy requires early recognition and prompt intervention with corticosteroids [11]. The incidence observed in our study highlights the need for heightened vigilance and patient education regarding the signs and symptoms of this syndrome.

The coagulopathy associated with APL, driven by tissue factor expression, annexin II-mediated hyperfibrinolysis, and proteolytic degradation of fibrinogen, creates a uniquely high-risk environment for bleeding complications. Previous studies have identified hyperleukocytosis as a factor predisposing to central nervous system involvement and hemorrhagic death [18]. Intracranial hemorrhage was the third most common cause of death, consistent with previous research identifying hemorrhagic complications as an important contributor to early mortality in APL [19].

The striking female predominance among deceased patients (78.6%) observed in our study has not been widely reported in the literature. This finding may reflect socioeconomic disparities affecting healthcare access and treatment adherence in our population, although the relatively small sample size precludes definitive conclusions. Confirmation in larger, prospective studies is warranted. Analysis of clinical and laboratory parameters revealed no statistically significant differences between patients with and without early death, although trends toward lower hemoglobin, lower platelet count, and higher blast percentage were observed in the deceased cohort. Previous studies have identified elevated WBC count and low platelet count as risk factors for ED, consistent with our observations [22]. Several additional blood chemistry parameters, including fibrinogen, creatinine, albumin, and C-reactive protein, have been associated with ED risk in other cohorts [23].

Several limitations of this study warrant acknowledgement. The retrospective design and relatively small sample size precluded multivariate analysis to identify independent risk factors for TRM. Causes of death were extracted from medical records rather than confirmed by autopsy, introducing potential misclassification. Additionally, the single-center design may limit generalizability to other populations and healthcare settings. Our findings have important implications for clinical practice. The persistently high ED rate in APL (28.6%) emphasizes the need for rapid diagnosis and immediate initiation of ATRA therapy in patients with suspected APL, even before molecular confirmation [24]. Healthcare providers who first encounter patients with suspected APL must recognize this as a medical emergency requiring urgent intervention. Enhanced supportive care measures, including aggressive management of coagulopathy, prompt treatment of infections, and early recognition of differentiation syndrome, are essential to reducing TRM.

Future research should focus on identifying novel risk factors for TRM, optimizing supportive care protocols, and developing predictive models to stratify patients according to their risk of early death. Nutritional support, infection prevention strategies, and central nervous system-directed prophylaxis represent potential areas for intervention. The development of validated tools to estimate TRM risk following intensive therapy would facilitate evidence-based treatment decisions and appropriate resource allocation [25]. Despite these limitations, this study provides real-world data from an underrepresented region and highlights clinically relevant patterns of early mortality in acute leukemia.

## Conclusions

Early TRM remains a significant cause of treatment failure in acute leukemia in real-world practice, with APL patients at particularly high risk. Sepsis and differentiation syndrome were the predominant causes of early death in our cohort, highlighting the importance of early recognition, prompt supportive care, and improved treatment pathways in resource-limited settings.
